# Effect of lacticaseibacillus paracasei PS23 on anxiety and sleep difficulties among office workers: a double-blind, randomized controlled pilot trial

**DOI:** 10.1186/s12991-025-00599-1

**Published:** 2025-10-09

**Authors:** Shu-I Wu, Kai-Liang Kao, Chen-Ju Lin, Ya-Ju Lin, I-Chieh Lin, Wan-Lin Chen

**Affiliations:** 1 Department of Medicine, MacKay Medical College, New Taipei City, Taiwan; 2 Section of Psychiatry and Suicide Prevention Center, MacKay Memorial Hospital, Taipei, Taiwan; 3 Department of Pediatrics, Far Eastern Memorial Hospital, New Taipei City, Taiwan; 4 Department of Neurology, Mackay Memorial Hospital, Taipei, Taiwan; 5 Department of Medical Research, Mackay Memorial Hospital, Taipei, Taiwan

**Keywords:** Probiotic, Office worker, Lacticaseibacillus paracasei PS23, Anxiety, Sleep

## Abstract

**Background:**

Over the past decade, sleep disturbances and stress have markedly increased within the general population. Conditions such as insomnia and poor stress management are linked to a range of physical and psychological symptoms, including cardiovascular diseases, sleep disorders, depression, or anxiety. Recent studies have shown that heat-treated *Lacticaseibacillus paracasei* PS23 (PS23) can alleviate anxiety in clinical nurses experiencing high stress. However, the potential benefits of live PS23- particularly its effects on sleep quality, anxiety, and stress- remain unexplored.

**Methods:**

This study recruited office workers aged 20–65 years who reported moderate to high levels of perceived stress. Participants in the intervention group received a daily dose of 20 billion colony-forming units of live PS23 for six weeks, while the control group received two placebo capsules containing microcrystalline cellulose powder. Outcomes were assessed at baseline and at the end of the trial, including measures of stress, anxiety, sleep quality, fatigue, activity levels, depression, and overall quality of life. Additionally, changes in salivary stress markers and antioxidant levels were evaluated.

**Results:**

Of the 50 eligible participants initially enrolled, 45 completed the six-week trial with high compliance (> 80%), including 24 in the PS23 group and 21 in the placebo group. Compared to the placebo group, the PS23 group showed statistically significant improvements in overall insomnia symptoms (group × time interaction, *p* = 0.011), sleep latency (*p* = 0.045), sleep maintenance (*p* = 0.002), and trait anxiety levels (group × time interaction, *p* = 0.044).

**Conclusion:**

This pilot randomized controlled trial suggests that live *L. paracasei* PS23 may offer meaningful improvements in sleep quality and anxiety reduction among office workers experiencing elevated stress levels.

**Registration:**

ClinicalTrials.gov Identifier: NCT05826704; registration date: 4/11/2023. *Exploring the Effects of Lactobacillus paracasei PS23 on Workplace-related Stress Symptoms Among Office Workers*; URL: https://clinicaltrials.gov/study/NCT05826704

## Introduction

Anxiety arises when individuals face or anticipate stressful situations, often manifesting as feelings of nervousness, fear, worry, or apprehension. Those with a high anxiety trait are more likely to interpret situations as threatening or challenging, and typically respond with heightened fear or nervousness [1]. Between 1990 and 2019, the global prevalence of anxiety disorders rose by over 55% [2]. According to the Global Burden of Disease Study 2019, approximately 301 million people- representing 4.05% of the world’s population- were affected by anxiety disorders. Notably, anxiety disorders rank as the sixth leading cause of disability-adjusted life-years (DALYs), accounting for up to 23.7 million DALYs among all mental health disorder [3]. Research highlights a reciprocal relationships between stress, anxiety, and sleep disturbances [4]. Excessive anxiety can trigger a wide range of physical and emotional symptoms, including headaches, dizziness, chest pain, muscle tension, irritability, poor concentration, and an increased risk of other health issues [1]. Sleep problems are particularly common, with nearly 50% of individuals with anxiety reporting difficulties in falling or staying asleep [1, 5, 6].

Stress is defined as a state of worry or mental tension triggered by challenging circumstances [7]. Over the past decade, stress levels among employees have risen significantly [8]. This increase is closely linked to the growing prevalence of anxiety, depression, and sleep disorders [9]. Adequate sleep plays a crucial role in mitigating stress and enhancing mood, cognitive function, and overall health. While external stressors—such as those related to work and the environment—may be difficult to control directly, it is important to explore innovative approaches to managing sleep disturbances that may be stress-related. This need is especially pressing given the global rise in sleep disorders [10–12].

The prevalence of insomnia and sleep-related issues has risen sharply- from 11.9% in 2002 to approximately 25% of the global population- highlighting a growing worldwide concern [10, 13, 14].The economic burden of these conditions is substantial, with annual healthcare costs reaching up to US$100 billion [15]. Notably, indirect costs- such as reduced work productivity, increased healthcare utilization, and a higher incidence of accidents- often exceed direct medical expenses [16]. Stress is a key contributor to sleep disturbances, as it can trigger physiological changes that interfere with both falling asleep and staying asleep. These changes may include elevated heart rate, muscle tension, and disruptions in digestive function. When stress becomes chronic, it can further degrade sleep quality and structure, reducing the amount of deep sleep and rapid eye movement (REM) sleep—both essential for physical and mental restoration. is a key contributor to sleep disturbances, as it can trigger physiological changes that interfere with both falling asleep and staying asleep. These changes may include elevated heart rate, muscle tension, and disruptions in digestive function. When stress becomes chronic, it can further degrade sleep quality and structure, reducing the amount of deep sleep and rapid eye movement (REM) sleep—both essential for physical and mental restoration [17].

Probiotics, particularly psychobiotics, have emerged as promising interventions for alleviating anxiety [10, 18, 19] and sleep disturbances by modulating the gut-brain axis [10]. These effects are thought to be mediated through several interconnected biological pathways. For instance, psychobiotics may influence central nervous system function by altering the production and availability of neurotransmitters of serotonin, dopamine and gamma-aminobutyric acid (GABA), which are critical for mood regulation and sleep. Significant increases in sleep quality, accompanied by reductions in anxiety and depressive symptoms, were observed in a randomized controlled trial (RCT) examining the intake of probiotic NVP-1704 mixed with *Lactobacillus reuteri* (NK33) and *Bifidobacterium adolescentis* (NK98) for eight weeks [18]. Similarly, Nishida et al. reported improvements in sleep quality and reductions in sleep problems after 5–12 weeks of *Lactobacillus gasseri* administration [20, 21]. Additional anti-inflammatory effects by reducing pro-inflammatory cytokines that might mitigate stress-induced neuroinflammation have also been reported [22]. Despite these promising mechanisms, clinical outcomes have varied across studies. For example, while some trials have reported significant improvements in sleep and mood following probiotic supplementation, others have found no notable effects on sleep, anxiety [23] or stress levels [24]. These discrepancies in findings may be attributed to variations in the probiotic strains used or the heterogeneity of the various participants included in the studies, encompassing healthy volunteers and clinical subjects with diagnoses of depression or anxiety.

In our previous randomized controlled trial, we demonstrated thatheat-treated *Lacticaseibacillus paracasei* PS23 (HT-PS23) could effectively reduce anxiety levels among clinical nurses experiencing high stress [24]. However, the potential benefits of live PS23—hereafter referred to as"PS23"—have not yet been investigated, particularly in relation to its anxiolytic, anti-stress, or sleep-enhancing effects. To address this gap, we conducted a double-blind, randomized, placebo-controlled trial over six weeks to evaluate the efficacy of live PS23 in alleviating anxiety and other mood-related symptoms, as well as improving sleep quality and cognitive function. The study focused on general workplace employees who reported experiencing high levels of perceived stress.

## Methods

### Participants

This is a double-blind, randomized, placebo-controlled (RCT) study. We collaborated with a technology company in Northern Taiwan. After a detailed explanation session, office workers aged between 20 to 65 years and perceived themselves to be in moderate to high stress conditions were recruited. Those [1] having antibiotics treatments in the recent one month; [2] have taken any kind of probiotic products in forms of powder, capsule, or tablets in recent two weeks (not including Yakult or yogurt); [3] have been diagnosed with cancer; [4] are allergic to lactic acid bacteria; [5] are taking medications for any acute diseases or sleep disorders; [6] currently pregnant or lactation; [7] not suitable to participate judged by the Principal Investigator were excluded. Participants could opt out of the trial at any time if they had side effects (e.g., bloating or diarrhea) or did not want to continue participating.

### Study process

An independent researcher used a random number generator (www.randomizer.org) to assign participants to either the PS23 or placebo group using block randomization (block size = 4). To ensure balanced gender representation, random sampling was stratified by gender. Blind assessors then invited participants from each group to sign informed consent forms, with group allocation concealed from the principal investigator, assessors, and laboratory staff. Eligible participants began the six-week trial by signing the informed consent during the enrollment visit and undergoing baseline neuropsychological assessments (Visit 1, V1). Saliva samples were also collected at this time. Participants were then randomly assigned to receive either PS23 or placebo, administered as two capsules once daily for six weeks. At the end of the trial (Visit 2, V2), participants completed a second round of neuropsychological evaluations and provided another saliva sample. Any remaining capsules were returned at this final visit. The study was approved by the Institutional Review Board of Mackay Memorial Hospital (IRB No. 22CT044be).

### Materials

The investigational probiotic in this study, *L. paracasei* PS23, has demonstrated potential benefits in improving anxiety and cognitive functions in animal and human studies [22, 24, 25]. The PS23 capsules were manufactured and supplied by the Bened Biomedical Co., Ltd. Each capsule contained *L. paracasei* PS23 powder and is equivalent to 10 billion Colony Forming Units (CFU). *L. paracasei* has been approved by the Taiwan Food and Drug Administration to be taken as an edible food supplement. Participants in the placebo group received capsule filled with microcrystalline cellulose powder, which were identifcal in appearance, size, color, and taste to the PS23 capsules, ensuring blinding integrity. Throughout the trial, participants were closely monitored for any adverse effects. No intervention-related adverse events were reported, indicating that the treatment was well tolerated.

#### Neuropsychological measures

##### The state and trait anxiety index (STAI):

The STAI is a 40-item self-report questionnaire that distinguishes between state anxiety (temporary emotional state) and trait anxiety (general tendency to experience anxiety). It is widely used in both clinical and research settings to evaluate anxiety levels and coping mechanisms [24, 26–28]. The Chinese version of the STAI has good internal consistency and concurrent validity [27].

##### The insomnia severity index (ISI)

The ISI is a 7-item self-report measure of insomnia that assesses the severity of insomnia symptoms, including difficulty falling asleep or maintain sleep, satisfactions, or impacts on daytime functioning [29, 30]. The Chinese version of the ISI demonstrated satisfactory psychometric properties. The Chinese version has shown excellent psychometric properties, with high discriminant validity (AUC > 0.85) and sensitivity and specificity exceeding 90% for identifying individuals with insomnia [31].

##### The perceived stress scale (PSS)

The PSS is a 14-item self-report measure that evaluates the degree to which individuals perceive their lives as stressful or overwhelming. [32] The Chinese version maintains a factor structure consistent with the original and has demonstrated satisfactory psychometric reliability and validity [33, 34].

##### The job stress scale (JSS)

The JSS is a 38-item self-report instrument developed to assess job-related stress. [35] We use the JSS Chinese version translated by the Ministry of Labor in Taiwan.[36] It contained questions that assess how much stress one feels at work, how satisfied one is with one’s life, how well one’s connections with others are, and how well one feels overall. We hypothesized that the job burden in office workers would remain unchanged before and after the intervention.

##### The patient health questionnaire-9 (PHQ-9)

The PHQ-9 is a widely used 9-item screening tool for depression. A cutoff score above 10 yields a sensitivity of 86% and specificity of 94% for detecting major depressive disorder. The Chinese version is validated for use in primary care and research settings [37].

##### Brief fatigue index (BFI)

The BFI is a 9-item measure that assesses the severity and impacts of fatigue[38] It is commonly used in clinical and research contexts to screen for fatigue and monitor treatment outcomes. The Chinese version has demonstrated high internal reliability (Cronbach’s α = 0.90–0.92) and moderate convergent validity. It is commonly used in clinical and research contexts to screen for fatigue and monitor treatment outcomes. The Chinese version has demonstrated high internal reliability (Cronbach’s α = 0.90–0.92) and moderate convergent validity [39].

### The international physical activity questionnaire (IPAQ)

The IPAQ assesses the frequency, intensity, and duration of physical activity across various domains (work, transport, leisure, and household) over the past seven days [40, 41]. The Chinese version of the IPAQ has acceptable psychometric properties [42].

### Quality of life enjoyment and satisfaction questionnaire short form (Q-Les-Q Sf)

This 16-item questionnaire evaluates satisfaction and enjoyment across multiple life domains, including physical health, mood, work, leisure, and social relationships [43]. The Mandarin-translated version of Q-LES-Q has been proofed to have high test–retest reliability (ICC = 0.75) and internal consistency (Cronbach’s α = 0.87) [44].

### Visual analog scale (VAS) of gastrointestinal symptoms

A 10-point Visual Analog Scale was employed, as in our earlier investigations [24, 45], to assess any gastrointestinal discomforts and track changes over time.

### The patient global impression scales of improvement (PGI-C)

The PGI-C is a patient-reported outcome measure used to assess perceived improvement following an intervention. It has demonstrated good reliability, validity, and correlation with clinician-rated outcomes [46].

### The objective neuropsychological assessment

#### The color trails test (CTT) 1,2

The CTT is a neuropsychological test that measure attention, mental flexibility, and processing speed [47]. With CTT1 evaluating attention and processing speed and CTT2 evaluating executive functioning and cognitive flexibility[48], the Chinese version of CTT demonstrated good reliability and validity in Chinese adults [49, 50].

### Saliva biomarkers

Saliva levels of cortisol, α-amylase [51], IgA [52], lactoferrin [53], and lysozyme [52] were examined because past literature has shown that they may be potential biomarkers associated the effects of probiotics in reducing stress, anxiety, or fatigue [54] An electrochemiluminescence immunoassay kit (Elecsys Cortisol, Roche Diagnostic, Germany) was used to measure the level of saliva cortisol. Enzyme- linked immunosorbent assay kits (produced by Immuno-Biological Laboratories, Inc., USA; Germany; and Assaypro LLC, USA) were used to measure levels of salivary α-amylase, IgA, lactoferrin, and lysozyme. The steps were all completed in accordance with the manufacturer’s instructions.

### Statistical analysis

To compare the difference between the two groups at baseline, Chi-squared test, t-test, ANOVA, or Mantel–Haenszel analyses were selected according to characteristics of the data and the purpose of the study. For each variable, the Shapiro–Wilk test was performed to examine the normality [55]. For categorical and continuous variables, the baseline characteristics and changes over time between V2 and V1 of the PS23 and the placebo groups were compared using the Pearson Chi-Squared or repeated measure ANOVA.

With estimations from previous studies and an anticipated mean of PSS being decreased by at least 20%, an α error of 0.05, and a power (1-β) of 0.80[56], and taking into account the 8% drop out rate from our prior trials [24, 45], the minimum required sample size was 15 for each group. Statistical analysis was performed using SPSS version 18.0 and Prism version 4.0 for GraphPad graphs. A p-value is defined below 0.05.

## Results

**Fig. 1:**
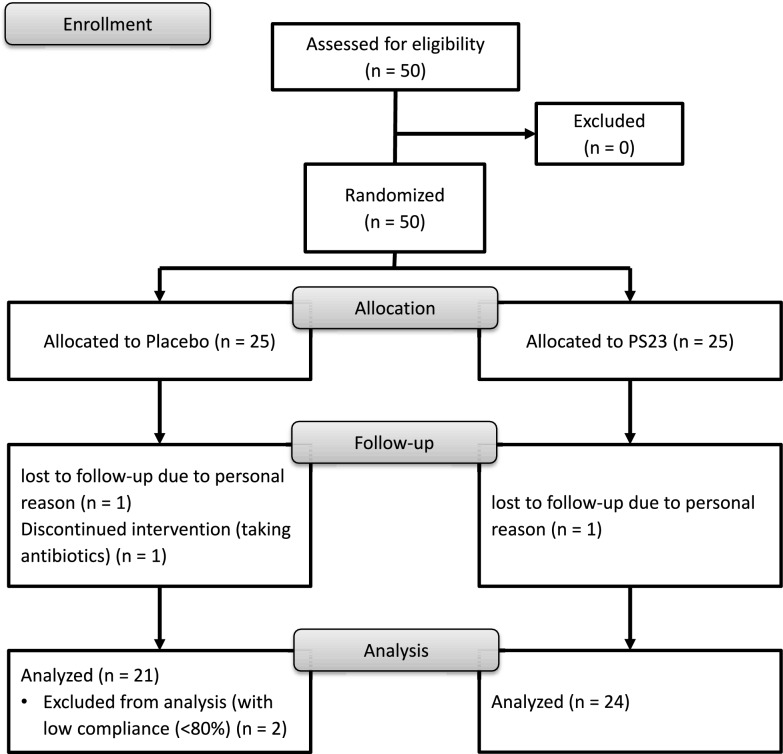
Study process

Figure 1 outlines the study flow. A total of 50 eligible office workers were invited to participate, all of whom met the inclusion criteria and provided informed consent. Participants were then randomly assigned to either the PS23 or placebo group. During the trial, one participant from each group withdrew due to international business travel. An additional participant from the placebo group discontinued participation after taking antibiotics during the intervention period. At the endpoint assessment, data from two more participants in the placebo group were excluded due to low compliance, as they returned 54% and 26% of their capsules, respectively. As a result, 24 participants in the PS23 group and 21 in the placebo group were included in the final analysis. The overall dropout rate was 10% (n = 5).

### Baseline evaluations

Table 1 presents the baseline comparisons between the PS23 and placebo groups. No statistically significant differences were found in demographic characteristics or in the mean scores of key measures, including the Perceived Stress Scale (PSS), Insomnia Severity Index (ISI), State-Trait Anxiety Inventory (STAI), Patient Health Questionnaire-9 (PHQ-9), Brief Fatigue Inventory (BFI), gastrointestinal symptom ratings, Quality of Life Enjoyment and Satisfaction Questionnaire (Q-LES-Q), International Physical Activity Questionnaire (IPAQ), and the Color Trails Test (CTT). These findings indicate that both groups were comparable at baseline across psychological, physical, and cognitive domains.

**Table 1:** Comparisons of baseline characteristics between the two groups in those completed the 6-week trial

	PS23 group (n = 24)		Placebo group (n = 21)		
	n	%	n	%	p-value
Gender					0.967
Male	9	37.5	8	38.1	
Female	15	62.5	13	61.9	
Marital status					0.315
Single	8	33.3	5	23.8	
Married	14	58.3	15	71.4	
Cohabit	0	0.0	1	4.8	
Divorced	2	8.3	0	0.0	
Children					0.124
With	13	54.2	16	76.2	
Without	11	45.8	5	23.8	
Education					0.469
Secondary	0	0.0	1	4.8	
High	1	4.2	0	0.0	
College/Vocational	7	29.2	5	23.8	
Bachelor	9	37.5	12	57.1	
Master’s Degree	6	25.0	3	14.3	
PhD/Doctorate	1	4.2	0	0.0	
Occupation Experience					0.311
< 6 years	1	4.2	0	0.0	
6 ~ 8 years	2	8.3	0	0.0	
8 ~ 10 years	1	4.2	3	14.3	
10 ~ 15 years	6	25.0	8	38.1	
> 15 years	14	58.3	10	47.6	
International Physical Activity Questionnaire (IPAQ)					0.578
Inactive	8	33.3	10	47.6	
Minimally Active	9	37.5	7	33.3	
HEPA Active	7	29.2	4	19.1	

	Mean	SD	Mean	SD	p-value
Age	44.6	10.9	41.2	7.5	0.236
Years of Education	16.0	2.1	15.5	1.9	0.395
Perceived Stress Scale (PSS)					
PSS-14 Total	36.3	7.6	39.7	7.5	0.133
PSS-10 Total	25.2	5.5	27.1	5.3	0.250
Job Stress Scale (JSS)					
JSS Stress	34.2	20.8	33.3	22.2	0.897
JSS Skill	25.9	5.4	25.1	6.1	0.654
JSS Autonomy	34.2	5.5	33.7	5.3	0.782
JSS Control	62.6	9.4	61.3	10.3	0.666
JSS Burden	61.3	9.6	65.8	8.8	0.112
JSS Supervisor	12.0	2.1	11.0	2.2	0.163
JSS Colleague	12.0	1.9	12.2	1.8	0.724
JSS Interpersonal	75.0	10.4	72.8	11.5	0.499
JSS Satisfaction	71.7	16.6	66.7	15.9	0.310
JSS Health	55.8	13.5	52.0	17.0	0.403
JSS Energy	50.6	16.0	44.0	13.8	0.150
JSS General	36.8	7.3	39.4	7.4	0.227
Color Trails Test (CTT)					
CTT1 Time	41.5	15.2	39.5	10.6	0.545
CTT1 Error	0.1	0.3	0.1	0.4	0.537
CTT1 Near-Misses	0.1	0.3	0.2	0.5	0.611
CTT1 Prompts	0.0	0.2	0.0	0.0	0.355
CTT2 Time	74.4	28.7	72.0	15.7	0.759
CTT2 Color Errors	0.1	0.3	0.2	0.4	0.315
CTT2 Number Errors	0.0	0.0	0.0	0.0	1.000
CTT2 Near-Misses	0.0	0.2	0.0	0.0	0.355
CTT2 Prompts	0.2	0.5	0.1	0.4	0.625
CTT Interference	81%	26%	88%	39%	0.369
Insomnia Severity Index (ISI)					
ISI Initial Insomnia	1.4	1.1	1.1	1.1	0.306
ISI Middle Insomnia	1.6	1.1	1.5	1.0	0.688
ISI Late Insomnia	1.2	1.3	1.3	1.0	0.745
ISI Total	11.2	6.7	10.0	4.7	0.513
State and Trait Anxiety Inventory (STAI)					
STAI State	43.7	9.6	45.6	9.9	0.516
STAI Trait	50.0	7.4	48.3	8.9	0.497
Visual Analog Scale for Gastrointestinal discomfort (VAS-GI)					
VAS-GI Total	20.7	17.0	22.8	16.0	0.674
Patient Health Questionnaire-9 (PHQ-9)					
PHQ-9 Total	7.8	4.4	8.1	4.7	0.772
Bried Fatigue Inventory (BFI)					
BFI Severity	5.1	2.4	5.1	2.3	0.983
BFI Interference	3.4	2.1	3.3	2.1	0.811
Quality of Life, Enjoyment, and Satisfaction Questionnaire (Q-LES-Q)					
Q-LES-Q Total	46.8	6.6	46.6	7.3	0.982
International Physical Activity Questionnaire (IPAQ)					
IPAQ MET Total	2547.4	4163.4	1971.9	2900.7	0.599
IPAQ MVPA	312.1	424.8	254.8	365.7	0.633

MET = Metabolic equivalent. HEPA = Health enhancing physical activity. Continuous variables were analyzed by independent t tests, Categorial variables were analyzed by Pearson Chi-Squared tests. Results were presented in *p <.05, **p <.01, ***p <.001

### Comparisons of between-group changes over time

Tables 2 and 3 present the results of the endpoint evaluation, providing a comparison of changes over time in neuropsychological measures between the PS23 and the placebo groups over the six-week trial.

**Table 2:** Changes of outcomes from pre- to post- tests between the two groups

	PS23 (n = 24)						Placebo (n = 21)							Group* Time
	V2			D (V2-V1)			V2				D (V2-V1)			
	Mean	SD		Mean	SD		Mean		SD		Mean	SD		p-value
Perceived Stress Scale (PSS)														
PSS-14 Total	32.2	8.7		-4.0	7.6		36.9		8.0		-2.8	4.6		0.522
PSS-10 Total	21.8	6.9		-3.4	5.9		24.3		5.9		-2.8	3.8		0.711
Job Stress Scale (JSS)														
JSS Stress	35.0	27.2		0.8	31.5		35.2		26.0		1.9	34.6		0.914
JSS Skill	24.6	4.4		-1.3	3.5		25.0		5.2		-0.1	4.8		0.328
JSS Autonomy	34.0	4.6		-0.2	4.6		34.7		5.0		1.0	4.9		0.435
JSS Control	61.0	7.5		-1.6	6.8		62.2		9.2		0.9	8.5		0.288
JSS Burden	57.7	6.7		-3.6	6.9		60.2		7.9		-5.1	11.3		0.588
JSS Supervisor	12.0	1.6		0.0	1.7		11.4		1.6		0.3	1.8		0.577
JSS Colleague	11.9	1.3		-0.1	1.6		12.5		1.7		0.3	1.7		0.409
JSS Interpersonal	74.7	7.4		-0.3	7.5		74.7		9.3		1.9	9.5		0.393
JSS Satisfaction	75.8	16.7		4.2	13.2		65.7		14.3		-1.0	14.8		0.226
JSS Health	64.3	15.9		8.5	11.6		60.0		16.8		8.0	17.3		0.909
JSS Energy	57.7	16.0		7.1	7.2		51.4		13.9		7.4	11.7		0.917
JSS General	40.2	8.1		3.5	5.9		39.0		6.1		-0.5	7.5		0.055
Color Trails Test (CTT)														
CTT1 Time	37.8	14.7		-4.2	9.0		33.5		7.0		-6.0	9.1		0.512
CTT1 Errors	0.0	0.0		-0.1	0.3		0.0		0.2		-0.1	0.4		0.913
CTT1 Near-Misses	0.0	0.2		-0.1	0.4		0.0		0.2		-0.1	0.6		0.688
CTT1 Prompts	0.0	0.2		0.0	0.3		0.0		0.0		0.0	0.0		1.000
CTT2 Time	66.5	22.9		-7.7	14.7		64.4		13.6		-7.6	16.3		0.985
CTT2 Color Errors	0.1	0.4		0.0	0.6		0.1		0.4		0.0	0.5		0.573
CTT2 Number Errors	0.0	0.2		0.0	0.2		0.0		0.0		0.0	0.0		0.355
CTT2 Near-Misses	0.0	0.2		0.0	0.3		0.0		0.2		0.0	0.2		0.546
CTT2 Prompts	0.1		0.4	-0.1		0.4		0.1		0.3	0.0		0.4	0.765
CTT Interference	80%		27%	2%		31%		94%		28%	6%		39%	0.845
Insomnia Severity Index (ISI)														
ISI Initial Insomnia	0.7		0.7	-0.7		0.8		0.9		1.0	-0.2		0.6	0.045*
ISI Middle Insomnia	0.8		0.9	-0.8		0.8		1.5		0.8	0.0		0.8	0.002**
ISI Late Insomnia	0.8		1.0	-0.3		0.7		1.2		1.0	0.0		1.1	0.362
ISI Total	7.0		4.9	-4.0		3.8		9.0		4.3	-1.0		3.8	0.011*
State and Trait Anxiety Inventory (STAI)														
STAI State	40.0		10.1	-4.6		7.4		44.0		10.0	-1.7		6.6	0.179
STAI Trait	46.1		8.3	-3.9		4.7		47.1		8.8	-1.2		3.9	0.044*
Visual Analog Scale for Gastrointestinal Discomfort (VAS-GI)														
VAS-GI Dry Mouth	3.7		2.6	-0.9		2.9		3.5		2.7	-0.4		2.8	0.614
VAS-GI Swallowing	1.0		2.1	-0.3		1.8		0.4		0.7	-0.4		1.0	0.865
VAS-GI Appetite	0.8		1.2	-1.0		1.9		1.2		1.6	-0.5		1.4	0.281
VAS-GI Nausea & Vomiting	0.6		1.6	-0.4		2.6		0.7		1.1	0.0		1.0	0.432
VAS-GI Bloating	2.8		3.3	-0.5		2.8		3.0		2.7	-1.4		2.6	0.273
VAS-GI Stomachache GERD	2.0		3.0	-0.2		2.0		1.4		1.5	-1.3		3.0	0.133
VAS-GI Upper Abdomen	1.0		2.1	0.3		1.7		1.5		2.3	0.0		2.5	0.696
VAS-GI Lower Abdomen	1.0		2.1	0.5		1.6		1.5		2.1	0.3		2.0	0.731
VAS-GI Constipate	2.3		3.0	-0.7		3.3		2.6		2.8	-1.1		2.4	0.652
VAS-GI Diarrhea	1.5		2.0	-0.7		2.2		2.5		2.9	0.1		2.2	0.220
VAS-GI Total	16.7		18.4	-3.9		12.3		18.2		12.1	-4.6		12.4	0.861
Patient Health Questionnaire-9 (PHQ-9)														
PHQ-9 Total	5.3		4.8	-2.5		2.6		6.6		4.2	-1.5		3.1	0.277
Brief Fatigue Inventory (BFI)														
BFI Severity	3.5		2.8	-1.6		1.6		3.7		2.3	-1.4		2.1	0.751
BFI Interference	2.2		2.2	-1.2		2.0		2.4		1.6	-0.9		1.9	0.535
Quality of Life, Enjoyment, and Satisfaction Questionnaire (Q-LES-Q)														
Q-LES-Q Total	49.9		7.6	3.3		6.6		48.7		7.1	2.0		6.1	0.531
International Physical Activity Questionnaire (IPAQ)														
IPAQ MET Total	3438.4		5036.1	891.0		2395.5		3670.4		7124.7	1698.5		5560.4	0.521
IPAQ MVPA	446.9		721.7	134.8		459.4		481.9		911.4	227.1		728.5	0.609

MET = Metabolic equivalent. HEPA = Health enhancing physical activity. Continuous variables were analyzed by repeated measure ANOVA. Results were presented in *p <.05, **p <.01, ***p <.001

**Table 3:** Comparison of PGI-C between the two groups

	PS23 (n = 24)		Placebo (n = 21)		p-value
	Mean	SD	Mean	SD	
Patient’s Global Impression of Change (PGI-C)	3.6	0.7	3.7	6.0	0.655

Continuous variables were analyzed by independent t test. Result was presented in *p <.05, **p <.01, ***p <.001

**Fig. 2:**
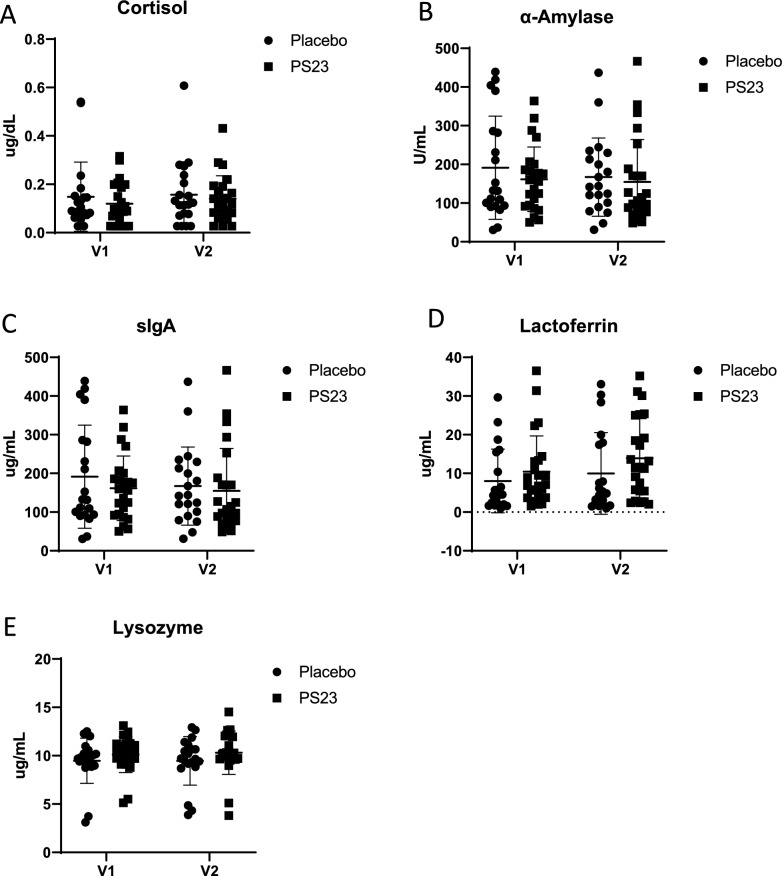
Saliva markers before and after the trial. Concentrations of salivary biomarkers in participants treated with placebo orPS23. Saliva samples were collected at the start of the study (V1) and at the end of the 6-weekintervention (V2). (**A**) Salivary cortisol, (**B**) α-amylase, (**C**) IgA, (**D**) lactoferrin, and (**E**) lysozymeconcentrations are shown for each participant in both the placebo and PS23 groups at both timepoints. Each dot represents a single participant's measurement

Significant group × time interactions were observed for STAI-trait (p = 0.044) and ISI (p = 0.011), indicating differential improvements between groups. Further analysis of the ISI revealed that the PS23 group experienced significantly greater improvements in difficulty falling asleep (p = 0.045) and difficulty staying asleep (p = 0.002). Although both groups showed significant improvements in several measures—including PSS total scores, job burden, JSS psychological health, JSS energy level, STAI-state and trait, PHQ-9, Q-LES-Q, fatigue, activity level, CTT1, CTT2, and gastrointestinal symptoms—no significant group × time interactions were found for these outcomes, suggesting similar improvements across both groups. Additionally, PGI-C scores did not differ significantly between the PS23 and placebo groups (Table 3), and salivary biomarkers showed no significant changes before and after the trial (Fig. 2).

## Discussion

This study represents the first pilot trial to explore the potential impact of a six-week probiotic *Lacticaseibacillus paracasei* PS23 intake in improving mental health and psychological conditions among office workers with perceived high stress levels. Employing a double-blind, randomized, placebo-controlled design, we observed that the use of PS23 was associated with improvements in trait anxiety and sleep quality after the six-week trial. The changes over time differed significantly between the PS23 and placebo groups suggesting that PS23 may offer distinct benefits in mitigating anxiety and sleep disturbances under stress.

The anxiolytic effects of PS23 observed in this study are consistent with previous findings in clinical populations, such as nurses under high stress. These effects may reflect a shift toward more adaptive stress responses, potentially mediated by gut-brain axis mechanisms [24]. One proposed pathway involves the modulation of neurotransmitter levels. Animal studies have shown that PS23 administration increased brain-derived neurotrophic factor (BDNF), serotonin, dopamine, and other monoamines [25]- key regulators of mood, emotional processing, and neuroplasticity [25] [22]. BENF, in particular, plays a critical role in synaptic plasticity and resilience to stress, and its upregulation has been associated with antidepressant and anxiolytic effects. Given that approximately 90% of serotonin is produced in the gastrointestinal tract, the gut microbiota can significantly influence central nervous system function. Through the activation of the enteric nervous system and vagus nerve, microbial metabolites and neuroactive compounds can provide signals to the brain, modulating emotional and behavioral responses. This bidirectional communication- often referred to as the microbiota-gut-brain axis- is increasingly recognized as the key mediator in the pathophysiology of anxiety and depression [57, 58].

Moreover, PS23 may influence the synthesis and release of gamma-aminobutyric acid (GABA), an inhibitory neurotransmitter that plays a central role in reducing neuronal excitability and promoting calmness. Certain strains of *Lactobacillus* have been shown to enhance GABAergic activity, which may contribute to the observed anxiolytic effects. These neurochemical changes, combined with anti-inflammatory and immunomodulatory actions, suggest that PS23 may exert its therapeutic effects through a multifaceted mechanism involving both neural and immune pathways [59].

One plausible explanation for the anxiolytic effects of PS23 is its potential role in mitigating stress-induced inflammatory responses [60]. Long-term stress has been suggested to affect blood corticosterone levels. Moreover, an increased inflammatory state in the gastrointestinal tract may be accompanied by the infiltration of monocytes or neutrophils alongside increased enteral permeability, leading to the penetration of bacteria or large molecules [25, 61] [22].Bacterial dysbiosis may further stimulate dysfunctional systemic immune responses, contributing to central nervous system inflammation [62]. Animal studies have suggested that PS23 may help counteract these effects. For instance, in mice exposed to early-life separation anxiety, PS23 administration was found to reduce serum corticosterone levels and increase interleukin-10 levels, an anti-inflammatory cytokine. These physiological changes were associated with improvements in anxiety-related behaviors, supporting the hypothesis that immune modulation and anti-inflammatory mechanisms may underlie PS23’s anxiolytic effects [22]. While these findings are promising, further research is needed to confirm the impact of *L. paracasei* PS23 on anxiety in humans. Larger clinical or subclinical trials, along with biological and neuroimaging studies, are essential to better understand the gut-brain axis and the mechanisms through which probiotics may influence mental health. [18].

The finding that PS23 significantly improved sleep latency and maintenance after the six-week trial is consistent with previous research suggesting that probiotics may enhanced sleep quality [20, 63, 64] [18, 65]. The reduction in the mean total ISI score in the PS23 group– from above the clinical threshold for insomnia to a level indicating no insomnia- is particularly encouraging. Although prior studies have not specifically investigated the effects of *L. paracasei* on sleep in individuals experiencing high stress levels, the observed improvements may be linked to changes in gut microbiota and the modulation of cytokines production. Cytokines such as interleukin-1βand tumor necrosis factor-α have been shown to promote non-REM sleep [66], suggesting a potential biological pathway through which probiotics may influence sleep. Additionally, the circadian rhythms of gut microbiota, their metabolites, and probiotics tend to align with the feeding/fasting cycle, which may help regulate sleep architecture and improve sleep duration and latency [67] [20]. Other mechanisms may include the synthesis and release of neurotransmitters—such as serotonin produced by enterochromaffin cells—which play a key role in sleep regulation, [10] as well as the enhancement of melatonin receptor expression [68]. *Lactobacillus* species have also been identified as potential contributors to gamma-aminobutyric acid production, which affects the central nervous system via the vagus nerve [69]. These combined effects may help explain the sleep-enhancing properties of PS23. However, further research is needed to confirm these mechanisms and to explore the potential of *L. paracasei* in improving sleep quality. Larger clinical trials and studies incorporating biological and neuroimaging data would be valuable in elucidating the gut-brain interactions involved.

Consistent with our earlier findings, the lack of significant changes in gastrointestinal symptoms between groups before and after the intervention suggests that oral administration of L. paracasei PS23 capsules is both acceptable and well tolerated [24]. However, these results contrast with previous studies that reported potential therapeutic benefits of probiotics in reducing cortisol levels, perceived stress, cognitive impairments, and symptoms of depression and fatigue [23, 65, 70] [71]. These discrepancies may be attributed to differences in probiotic strains, duration and timing of administration, or variations in the target populations and diagnostic criteria. Additionally, other confounding factors not accounted for in this study—such as individual microbiota profiles or genetic interactions—may also influence outcomes and should be considered in future research.

Although several validated scales in this study revealed statistically significant improvements in anxiety and sleep parameters following PS23 supplementation, the Patient Global Impression of Change (PGI-C) did not show significant differences between the intervention and placebo groups. This finding suggests that, despite measurable improvements in specific symptoms, participants may not have perceived a substantial overall change in their condition. There are several possible explanations for this discrepancy. First, the PGI-C is a single-item global measure that captures subjective impressions of change, which may be influenced by expectations, personality traits, or subtle symptom fluctuations that are not easily detected over a short intervention period. Second, baseline functioning of this study population may have been sufficiently high that even meaningful improvements in specific domains did not translate into a strong sense of overall change.

Additionally, the placebo effect may have played a role, particularly that the PGI-C relying heavily on self-reported measures. Participants in both groups may have experienced perceived benefits simply from participating in a structured wellness study. Finally, the six-week duration may have been too short for participants to recognize broader improvements in well-being, especially if changes were gradual or subtle. Future studies should consider complementing PGI-C with qualitative interviews or longer follow-up periods to better capture perceived global changes and contextualize symptom-specific improvements. [18].

## Strengths and limitations

The primary strengths of this study lie in its prospective, double-blind RCT design, aimed at investigating the potential effects of PS23 in a homogenous group of adults experiencing high levels of perceived stress. A key advantage was the comprehensive evaluation conducted at both baseline and endpoint of the six-week trial, encompassing psychological measures (stress, anxiety), cognitive assessments (attention and executive function), and biological markers (salivary stress indicators). Participant compliance with the intervention was also satisfactory, further supporting the reliability of the findings. However, several limitations should be noted. The most significant was the small sample size, which limits statistical power and generalizability. Larger-scale studies are needed to confirm the effectiveness of PS23 in reducing stress and anxiety. Despite this, the pilot study offers valuable insights into the acceptability and tolerability of probiotics for improving sleep among stressed individuals. Second, the generalizability of the findings may be constrained by the specific demographic and occupational characteristics of the study population. Results may not apply to individuals with different backgrounds or working conditions. Third, the single-phase intervention design may not capture long-term biological and psychological changes. Future studies should consider extended follow-up periods to assess sustained effects. Fourth, while the Chinese Job Stress Scale (JSS) used in this study was translated by the Taiwanese Ministry of Labor and is commonly applied in occupational settings, it lacks formal psychometric validation in peer-reviewed literature. This may limit the interpretability and generalizability of job stress-related findings. Future research should consider using validated instruments or conducting psychometric evaluations of locally adapted scales to ensure measurement accuracy and cross-study comparability. Finally, although this study included a range of assessments related to mood, physical activity, and biological markers of stress, inflammation, and antioxidant status, it did not examine microbiota composition or autonomic nervous system activity. Additional factors such as dietary habits and genetic variations, which may act as confounders, were also not explored. Future research should incorporate dietary records, objective sleep measurements, and microbiome analyses to better understand the mechanisms underlying the observed effects and to clarify the gut-brain connection.

## Conclusion

This pilot randomized controlled trial demonstrated the potential benefits of administering the psychobiotic *Lacticaseibacillus paracasei* PS23 in alleviating trait anxiety and enhancing sleep latency and maintenance among individuals experiencing high levels of perceived stress. The study also confirmed the safety and feasibility of PS23 supplementation. However, further research is needed to investigate whether the observed effects are mediated through interactions between gut microbiota and the gut-brain axis, which may serve as a key regulatory pathway.

## Data Availability

No datasets were generated or analysed during the current study.
